# Draft Genome Sequence of Vibrio jasicida 20LP, an Opportunistic Bacterium Isolated from Fish Larvae

**DOI:** 10.1128/MRA.00813-21

**Published:** 2021-11-04

**Authors:** Gracinda M. M. Sanches-Fernandes, Gianmaria Califano, Tina Keller-Costa, Sara Castanho, Florbela Soares, Laura Ribeiro, Pedro Pousão-Ferreira, Leonardo Mata, Rodrigo Costa

**Affiliations:** a Institute for Bioengineering and Biosciences, Instituto Superior Técnico, Universidade de Lisboa, Lisbon, Portugal; b Associate Laboratory Institute for Health and Bioeconomy, Instituto Superior Técnico, Universidade de Lisboa, Lisbon, Portugal; c Centre of Marine Sciences, University of Algarve, Faro, Portugal; d Institute for Inorganic and Analytical Chemistry, Friedrich-Schiller-Universität Jena, Jena, Germany; e Portuguese Institute for the Ocean and Atmosphere, Aquaculture Research Station, Olhão, Portugal; f Lawrence Berkeley National Laboratory, Department of Energy-Joint Genome Institute (DOE-JGI), UC Berkeley, Berkeley, California, USA; University of Delaware

## Abstract

We present the genome sequence of Vibrio jasicida 20LP, a bacterial strain retrieved from larvae of gilthead seabream (Sparus aurata), a highly valuable, model fish species in land-based aquaculture. Annotation of the V. jasicida 20LP genome reveals multiple genomic features potentially underpinning opportunistic associations with diverse marine animals.

## ANNOUNCEMENT

Vibrio jasicida is a relatively recently described bacterial species that belongs to the Vibrio harveyi clade and can be found in association with diverse marine animals, such as lobsters, gastropods, and fish (1). Vibrio jasicida (formerly classified as V. harveyi) has been proposed as the causative agent of vibriosis, leading to mortality rates of >75% for phyllosoma larvae of the packhorse rock lobster Jasus verreauxi (2), which raises concerns about the pathogenic potential of this species across a broad range of marine animals in both natural and built environments. To increase our understanding of the species’ aptitude to colonize, persist, and exploit animal hosts, here we report the genome sequence of V. jasicida strain 20LP.

V. jasicida 20LP was isolated after cultivation of microbial cell suspensions derived from gilthead seabream larvae on thiosulfate-citrate-bile salts-sucrose (TCBS) agar (Oxoid, USA) for 7 days at 22°C (3) and was classified by 16S rRNA gene sequencing using the sequence match tool of the RDP database (http://rdp.cme.msu.edu), as described previously (4). Prior to genome sequencing, DNA was obtained with the Wizard genomic DNA purification kit (Promega, USA) from a fresh culture prepared in marine broth for 2 days at 19°C (5). The Illumina Nextera XT DNA library preparation kit (insert size, ∼450 bp) was employed for library construction, and paired-end sequence reads (125 cycles) were generated on an Illumina HiSeq 2500 platform at BaseClear (The Netherlands). For all bioinformatic analyses, default parameters were used unless specified otherwise. FASTQ sequence files were created with the Illumina Casava pipeline v1.8.3. Thereafter, reads containing adapters and low-quality reads were removed using BBtools v38.86 (http://bbtools.jgi.doe.gov). The sequencing output was 467.4 Mb, comprising 3,709,520 reads of 126 bp. Genome assembly was performed with the *de novo* assembly option within the CLC Genomics Workbench v7.0.4, and the optimal *k*-mer size was automatically determined using KmerGenie v1.6213 (6). Table 1 displays the general features of the V. jasicida 20LP genome, which possesses 97.8% average nucleotide identity (ANI) with respect to the genome of the type strain V. jasicida LMG 25398 (GenBank accession number GCF_000400365.1), as assessed on the IMG/M platform v6.0 (7).

**TABLE 1:** General features of the Vibrio jasicida 20LP genome

Feature	Description
Strain	Vibrio jasicida 20LP
Host species	Sparus aurata
No. of reads	3,709,520
Read length (bp)	126
Genome size (Mb)	5.84
GC content (%)	45.0
Genome coverage (×)	80.1
No. of contigs	72
Contig *N*_50_ (bp)	125,793
Completeness (%)^*a*^	96.4
Contamination (%)^*a*^	3.6
GenBank assembly accession no.	GCF_903995475.1
SRA accession no.	ERR6053175
GenBank accession no.	NZ_CAJFAE000000000.1

a Completeness and contamination were estimated with the MiGA webserver (13).

Genome annotation was carried out with Rapid Annotation using Subsystem Technology (RAST) v2.0, using the RASTtk protocol (8), and the NCBI Prokaryotic Genome Annotation Pipeline (PGAP) v4.1 (9). The gene-calling data were as follows: total number of genes: RAST, 5,395; PGAP, 5,201; number of coding sequences (CDSs): RAST, 5,309; PGAP, 5,111; total number of RNA genes: RAST, 86; PGAP, 90; number of rRNAs: RAST, 4; PGAP, 4; number of tRNAs: RAST, 82; PGAP, 82. Sixty-two CDSs were classified into the virulence, disease, and defense subsystem within RAST, of which 47 CDSs were involved in resistance to antibiotics, toxic compounds, and bacteriocins; these include genes encoding proteins for copper homeostasis (13 CDSs), bile hydrolysis (2 CDSs), and resistance to cobalt-zinc-cadmium (5 CDSs), fluoroquinolones (2 CDSs), colicin E2 (bacteriocin) (2 CDSs), copper (13 CDSs), and chromium (1 CDS), as well as multidrug resistance efflux pumps (9 CDSs). Regarding invasion and intracellular resistance, 14 CDSs resembling the Mycobacterium virulence operon were found, with 6 and 3 CDSs involved in small subunit (SSU) and large subunit (LSU) ribosomal protein synthesis, respectively, 2 CDSs involved in DNA transcription, and 3 CDSs involved in quinolinate biosynthesis. As usual among *Vibrio* species (10–12), strain 20LP likely hydrolyzes chitin, as multiple chitinase-encoding genes were annotated in its genome, suggesting opportunistic behavior toward organisms that use chitin as a primary structural biopolymer.

### Data availability.

The genome sequence was deposited in the European Nucleotide Archive (ENA) under BioProject accession number PRJEB9149, BioSample accession number SAMEA7110814, GenBank assembly accession number GCF_903995475.1, and SRA accession number ERR6053175. The annotation reported in this study is available on the RAST platform for guest users under job number 877706, identification number 6666666.587973.
